# Robotic-assisted right upper lobectomy with systemic pulmonary vein anomaly: a case report

**DOI:** 10.1186/s13019-023-02474-0

**Published:** 2024-01-03

**Authors:** Wenwu Liu, Shaohua Xie, Kaixin Zhang, Yingzhi Zhao, Xin Gao, Wei Dai, Qiuling Shi, Bin Hu, Qiang Li, Xing Wei

**Affiliations:** 1 Department of Thoracic Surgery, Sichuan Clinical Research Center for Cancer, Sichuan Cancer Hospital & Institute, Sichuan Cancer Center, Affiliated Cancer Hospital of the University of Electronic Science and Technology of China, No. 55, Section 4, South Renmin Road, Chengdu, 610041 China; 2 Graduate School, Chengdu Medical College, Chengdu, Sichuan China; 3 State Key Laboratory of Ultrasound Engineering in Medicine, School of Public Health, Chongqing Medical University, Chongqing, China

**Keywords:** Lung cancer, Lobectomy, Pulmonary vein variation, Robot-assisted surgery, Three-dimensional reconstruction technology

## Abstract

**Background:**

While the role of low-dose computed tomography (CT) in lung cancer screening is established, its limitations in detailing pulmonary vascular variations are less emphasized. Three-dimensional reconstruction technology allows surgeons to reconstruct a patient’s bronchial and pulmonary vascular structures using CT scan results. However, low-dose CT may not provide the same level of clarity as enhanced CT in displaying pulmonary vascular details. This limitation can be unfavorable for preoperative detection of potential pulmonary vascular variations, especially in cases involving planned segmentectomy.

**Case Presentation:**

We report a case of a 58-year-old female with lung cancer, initially planned for Da Vinci robot-assisted thoracoscopic segmentectomy. Unexpectedly, during surgery, a pulmonary vein variation in the right upper lobe was discovered, leading to a change in the surgical method to a lobectomy. The patient had four variant right upper lobe veins draining into the superior vena cava and one into the left atrium. The surgery was complicated by significant bleeding and postoperative pulmonary congestion. Postoperative pathology confirmed adenocarcinoma.

**Conclusions:**

This case highlights the importance of meticulous intraoperative exploration, particularly in cases involving planned segmentectomy, as unexpected pulmonary vein variations can significantly affect surgical decision-making. While three-dimensional reconstruction based on preoperative CT data is a valuable tool, it may not capture the full complexity of the anatomical variations. We discuss potential preoperative imaging techniques, including contrast-enhanced CT and CT angiography, as methods to better identify these variations. The enhanced visualization provided by robot-assisted surgery plays a crucial role in identifying and adapting to these variations, underscoring the advantages of this surgical approach. Our report contributes to the existing literature by providing a detailed account of how these principles were applied in a real-world scenario, reinforcing the need for surgical adaptability and awareness of the limitations of low-dose CT in complex cases.

## Background

The use of low-dose computed tomography (CT) has made it easier to identify patients with early-stage lung cancer. In addition to identifying lung cancers, CT scans can occasionally reveal systemic pulmonary vein (PV) anomalies, which are rare but clinically significant due to their potential cardiopulmonary implications. These anomalies can range from asymptomatic variations to those causing significant hemodynamic changes, potentially impacting surgical planning and outcomes [1]. CT scan results can be used to create a three-dimensional reconstruction of a patient’s bronchial and pulmonary vascular structures [7]. However, the detection and detailed visualization of systemic vein anomalies can be challenging, particularly with low-dose CT scans [10, 11]. The quality of CT images significantly influences the results of three-dimensional reconstruction^11^.

Nonetheless, despite the reduction in radiation exposure in patients, low-dose CT may not provide the same level of clarity as enhanced CT in characterizing pulmonary vascular details [8]. This is particularly pertinent in the context of systemic PV anomalies, where detailed imaging is crucial to understand the potential cardiopulmonary implications of these variations. This limitation can be unfavorable for preoperative detection of potential pulmonary vascular variations, especially in cases involving planned segmentectomy [7, 9, 8]. To improve preoperative detection of these variations, contrast-enhanced CT or CT angiography could be considered, although their routine use may lead to increased radiation exposure and healthcare costs [10, 11].

We encountered a case in which the patient’s PV variation in the right upper lobe (RUL) was not identified preoperatively but was discovered during surgery. Consequently, we modified the surgical method from the planned Da Vinci robot-assisted thoracoscopic segmentectomy to a lobectomy. This case report aims to highlight a unique scenario where standard preoperative imaging failed to detect complex PV variations, emphasizing the need for surgical adaptability and the potential benefits of robotic-assisted surgery in managing such complexities.

## Case presentation

### Patient information

A 58-year-old female patient experienced a persistent cough for over a month after coronavirus disease infection. The patient had no relevant medical history, no history of smoking or alcohol consumption, and no family history of hereditary diseases. Upon admission, her blood pressure was 129/88 mmHg, height was 158 cm, and weight was 54 kg.

### Clinical findings

**Fig. 1:**
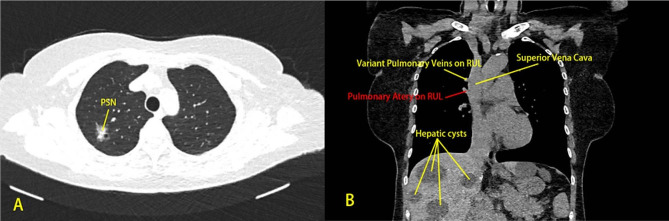
Pulmonary nodule on preoperative low-dose CT. PSN: Partial Solid Nodule, RUL: Right Upper Lobe

The patient underwent low-dose chest CT and abdominal ultrasonography at the outpatient clinic 3 weeks before admission. Low-dose lung screening CT showed a partial solid nodule in the posterior segment of the RUL of the lung. The nodule had a maximum and minimum diameter of 18.5 mm and 14.3 mm, respectively (Fig. 1A/B). The margins of the nodule were blurred, with small vacuoles and capillaries crossing. The abdominal ultrasound revealed multiple liver lesions, suspected to be hepatic cysts, with the largest one measuring approximately 7.8 × 6.7 × 7.2 cm in the right hepatic lobe. After admission, further preoperative assessments were completed. Electrocardiography revealed a heart rate of 71 beats per minute, indicative of a sinus rhythm. Pulmonary function testing indicated that the measured forced expiratory volume in 1 s (FEV1) was 2.23 L, with a measured/expected value ratio of 101.6%. The measured maximal voluntary ventilation (MVV) was 79.61 L/min, with a measured/expected value ratio of 89.9%. The measured diffusing capacity of the lungs for carbon monoxide (DLCO SB) was 8.06 mmol/min/kPa, with a measured/expected value ratio of 109.7%. Echocardiography revealed an ejection fraction (EF) of 73%, with only mild tricuspid valve regurgitation. Based on the CT images, we noticed PV variations in the RUL (Fig. 1B). The remaining positive findings included multiple hepatic cysts on abdominal ultrasonography, and mild tricuspid regurgitation on echocardiography.

### Diagnostic assessment

Considering the location, size, and nature of the pulmonary nodule [2], the fact that thoracoscopic surgery is less invasive than open surgery [3], and that segmentectomy preserves more pulmonary function than lobectomy [4], the initial preoperative planning was involving Da Vinci robot-assisted thoracoscopic resection of the posterior segment of the RUL. This choice was made to minimize the patient’s postoperative symptom burden while effectively addressing the nodule [13].

### Surgical intervention

We used a fourth-generation Da Vinci robotic system for this surgery. The patient was placed in the left lateral decubitus position. The robot’s 2nd (left) arm utilized bipolar coagulation forceps and entered the chest cavity through an operating port in the right 9th intercostal space. The robot’s 4th (right) arm was equipped with a monopolar coagulation hook and was entered through an operating port in the right 6th intercostal space. The robot’s 3rd arm was a thoracoscope for observation, which entered the chest through an observation port in the right 8th intercostal space. Simultaneously, an auxiliary incision approximately 4–5 cm in length was made approximately 3–4 cm below the front lower edge of the thoracoscopic observation port in the 8th intercostal space. The instruments were inserted through this incision.

**Fig. 2:**
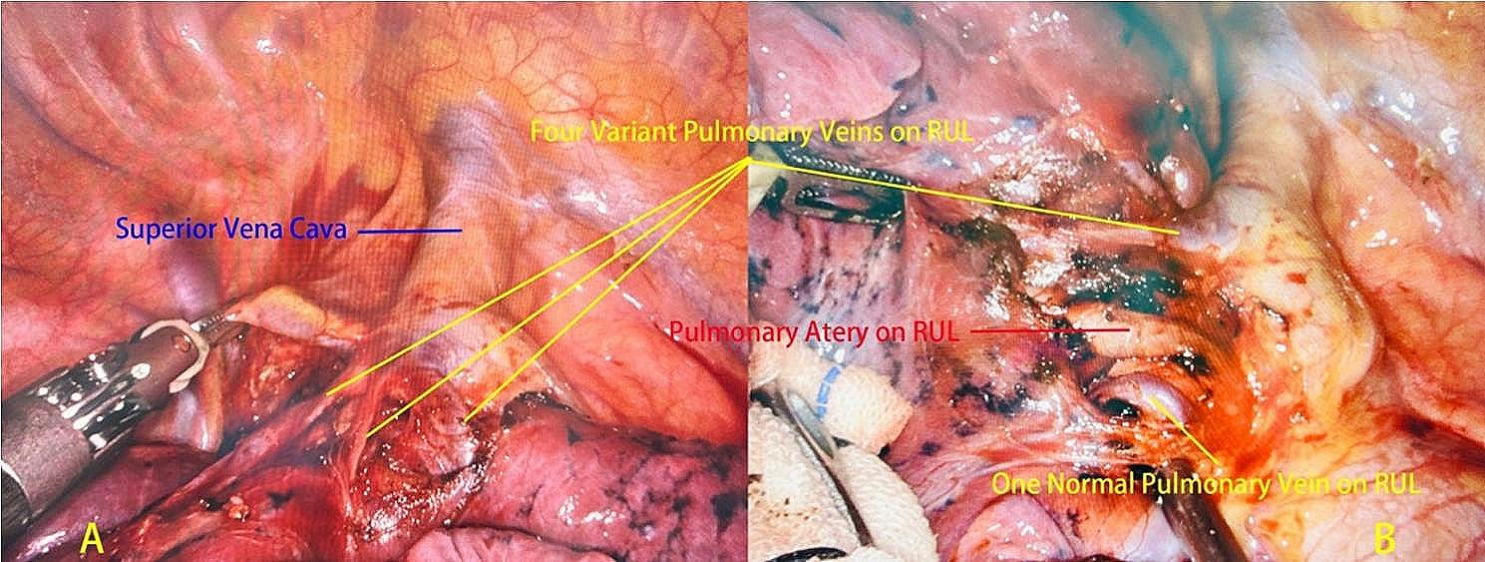
The variant RUL vein was discovered during the surgery. RUL: Right Upper Lobe

During anatomical dissection, we identified variations in the veins of the RUL. Upon careful examination, we found that four of these variant RUL veins drained directly into the superior vena cava, whereas one drained into the left atrium (Fig. 2A/B). These variations not only increased the complexity of performing a segmentectomy but also elevated the risk of intraoperative vascular incidents, leading to significant bleeding and postoperative pulmonary congestion. Thus, we decided to change the surgical method to a right upper lobectomy to minimize these risks.

We meticulously dissected and individually ligated the five variant RUL veins. During this process, we also carefully examined and managed the pulmonary arterial branches. These branches were found to be anatomically normal and were dissected with precision to ensure a clear surgical field and minimize the risk of intraoperative complications. Subsequently, we individually addressed the normal arteries and bronchi of the RUL and ultimately successfully removed the entire RUL. After extracting the specimen, we performed an intraoperative frozen-section pathological examination and definitive standard pathological examination of the nodule, confirming the adenocarcinoma diagnosis. Consequently, in accordance with the lymphatic drainage region of the RUL, we systematically cleared the lymph nodes in the 2nd, 4th, 7th, 10th, and 11th groups. The surgery took 80 min, with a minimal estimated blood loss of approximately 50 mL. The patient was awakened from the anesthesia and safely transferred back to the thoracic surgery ward.

### Follow-up and outcomes

Postoperatively, we administered prophylactic antimicrobial therapy using an intravenous infusion of 1 g of cefazolin every 8 h, initialized the afternoon of the second postoperative day. Additionally, we administered 3075AxaIU subcutaneous injections of nadroparin calcium once daily to prevent thrombosis until the fifth postoperative day. During hospitalization, we administered 0.3 g of acetylcysteine for nebulized inhalation twice daily. For pain management, we administered two tablets of sustained-release ibuprofen and codeine phosphate (each tablet containing 0.2 g and 13 mg of ibuprofen and codeine phosphate, respectively) as needed.

On the first postoperative day, a chest radiograph was obtained at the patient’s bedside. It revealed only a small amount of pleural effusion, which was attributed to postoperative changes. The first postoperative complete blood count showed a hemoglobin level of 118 g/L and a white blood cell count of 10.14 × 10^9^/L. The patient’s cardiac monitor and urinary catheter were removed on the morning of the first postoperative day. Remarkably, the patient had already begun ambulation and activity by the afternoon of the first postoperative day to facilitate postoperative recovery.

**Fig. 3:**
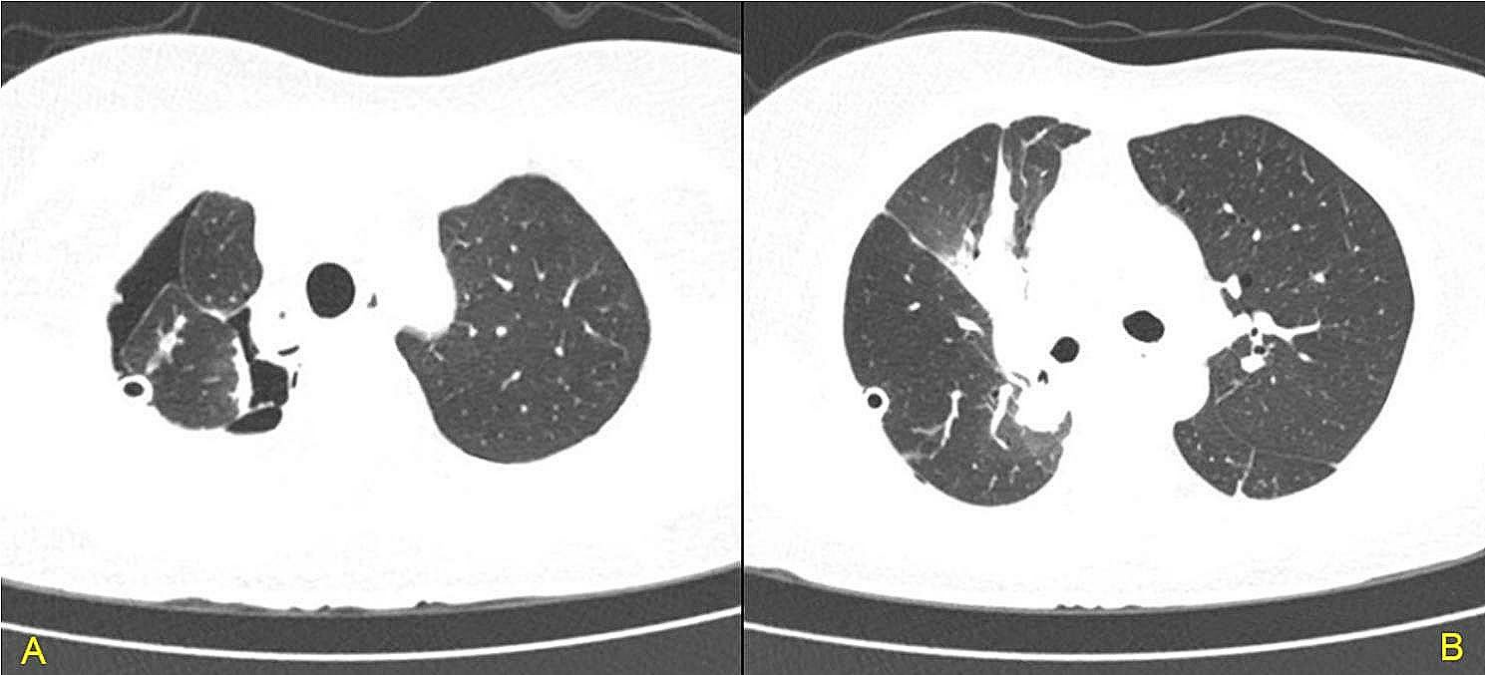
Plain chest CT on postoperative day 3

Due to the presence of a small amount of air leak within the closed chest drainage tube during coughing until the third postoperative day, we conducted a follow-up chest CT scan. CT results showed only a minimal amount of gas and fluid within the pleural cavity, without significant signs of pulmonary congestion or infection (Fig. 3A/B). The chest drainage volume was measured every 24 h postoperatively and decreased sequentially to 280, 200, 80, and 80 mL. Consequently, on the fifth postoperative day, we removed the closed chest drainage tube when there was no longer air leak during coughing. After tube removal, the patient did not report any specific discomfort and was discharged on postoperative day six. The definitive standard pathological examination of the resected lung tissue, measuring 10.5 × 8.2 × 1.7 cm and containing a nodule of 1.7 × 1.2 × 1.0 cm, showed no invasion into the visceral pleura. The nodule presented a gray-white and gray-brown appearance on its cut surface, characterized by a solid texture and moderate density. Immunohistochemical analysis yielded the following results: Ckpan (AE1/AE3) was positive, P63 showed partial positivity, TTF-1 and Napsin A were positive, while CK5/6 and TRIM29 were negative. These findings align with a diagnosis of adenocarcinoma, predominantly featuring an acinar pattern (approximately 95%) and a lesser extent of a micropapillary pattern (about 5%). The examination of all lymph nodes revealed no metastatic involvement, classifying the tumor as stage IA2 with a pathologic staging of pT1bN0M0.

**Fig. 4:**
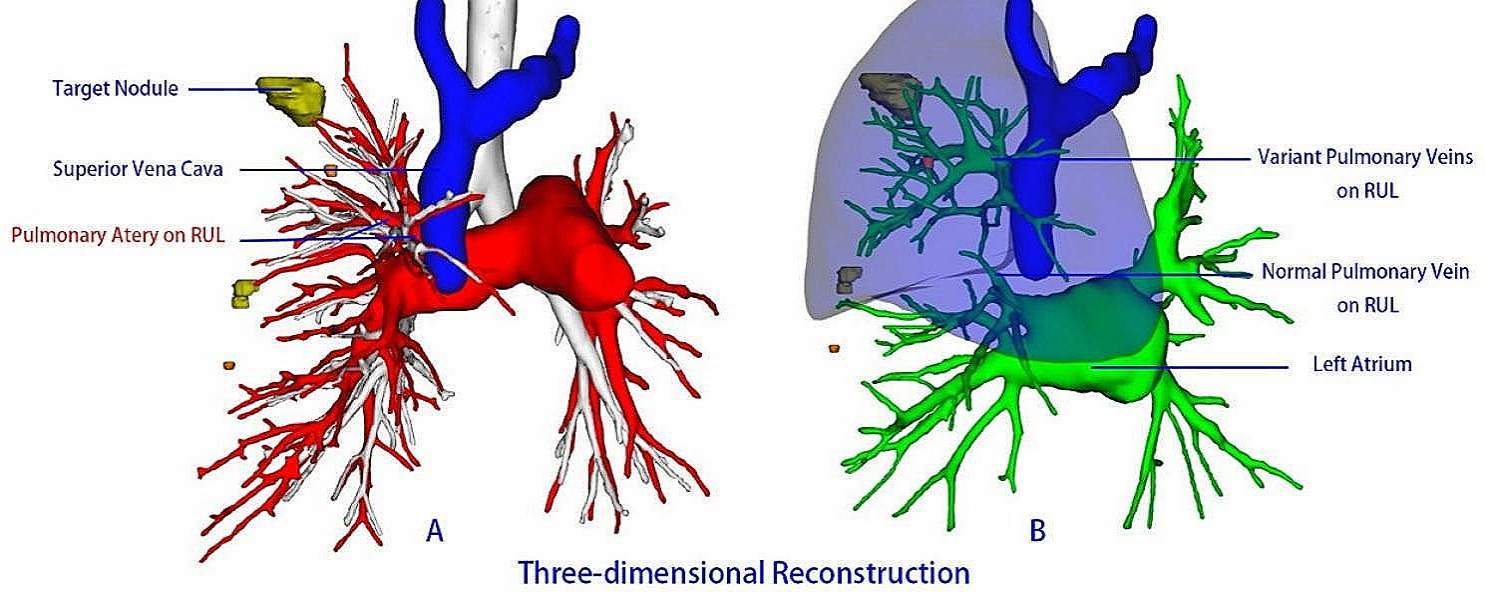
Three-dimensional reconstruction using preoperative low-dose chest CT scan. RUL: Right Upper Lobe

Since we discovered a variation in the patient’s RUL vein during surgery, we obtained the patient’s consent to conduct a complementary three-dimensional reconstruction postoperatively using preoperative low-dose chest CT imaging data. Although the three-dimensional reconstruction results did indicate the presence of a variation in the RUL vein, the four variant RUL veins observed during surgery were not clearly visualized. The three-dimensional reconstruction of the RUL (Fig. 4A/B) showed: (i) no variations in the bronchus, pulmonary artery, or their branches; (ii) the variant RUL vein drained into the superior vena cava, whereas the nonvariant vein drained into the left atrium.

## Discussion and conclusions

We encountered a patient with a RUL nodule who underwent Da Vinci robot-assisted thoracic surgery. The unexpected discovery of PV variation during surgery led us to choose a pulmonary lobectomy instead of a planned segmentectomy. This decision aimed to reduce potential postoperative complications associated with PV variations. The incidence of PV variations, such as the one encountered in our case, is relatively low, with studies reporting varying frequencies [1, 5]. These variations, while rare, can have significant hemodynamic implications, particularly in surgical contexts. In our patient, the direct drainage of the variant RUL vein into the superior vena cava could have led to altered hemodynamics, potentially contributing to the mild tricuspid regurgitation observed. Although some reports have been published, this anomaly is exceptional [1]. These unforeseen changes underscore the necessity of surgical adaptability and the importance of intraoperative decision-making [6]. Our case reinforces these well-known principles in the context of robotic-assisted thoracic surgery, providing a practical example of their application [12]. This case is significant as it illustrates the challenges surgeons may face with PV variations, which are not always detectable through standard preoperative imaging techniques. The enhanced visualization capabilities of the Da Vinci robotic system played a crucial role in identifying these variations, demonstrating the system’s value in complex thoracic surgeries [12].

We used three-dimensional reconstruction technology postoperatively, not preoperatively, to verify whether the variant RUL veins could be accurately displayed using low-dose CT-based three-dimensional reconstruction. The results showed that the reconstruction did not fully display these variations, revealing the limitations of low-dose CT in vascular imaging, particularly in displaying variant vessels. This finding emphasizes the need for vigilance when using low-dose CT for preoperative planning, especially when dealing with cases that may involve anatomical variations [8, 9, 10]. To enhance preoperative detection of such variations, contrast-enhanced CT or CT angiography could be considered [8]. These methods provide clearer visualization of vascular structures, potentially identifying variations not seen in low-dose CT. However, the routine use of these more detailed imaging techniques should be balanced against potential risks, such as increased radiation exposure and healthcare costs [11].

In the clinical environment in China, many patients undergo surgical planning and surgery based solely on preoperative low-dose CT. This may lead to an inadequate preoperative assessment of the actual vascular variations in patients [8]. Through this case report, we aim to remind clinicians of the limitations of low-dose CT in displaying vascular details when planning surgeries, particularly in cases that may involve anatomical variations. This highlights the importance of meticulous intraoperative exploration, as it can reveal unexpected anatomical variations that may significantly impact the surgical strategy [10]. One of the key advantages of robot-assisted surgery is enhanced visualization provided to the surgical team [12]. The high-definition 3D visualization system of the Da Vinci robot offers a magnified and detailed view of the surgical field, allowing precise identification of anatomical structures [12]. This level of clarity is invaluable for identifying unexpected PV variations during the procedure.

This case underscores the importance of meticulous intraoperative exploration, particularly in cases involving planned segmentectomy, as unexpected PV variations can significantly affect surgical decision-making. While three-dimensional reconstruction based on preoperative CT data is a valuable tool, it may not capture the full complexity of the anatomical variations. The enhanced visualization provided by robot-assisted surgery plays a crucial role in identifying and adapting to these variations, highlighting the advantages of this surgical approach. Surgeons must remain adaptable and prepared to modify their surgical method based on intraoperative findings to ensure the best patient outcomes.

## Data Availability

The datasets used are available from the corresponding author on reasonable request.

## Abbreviations

CT: Computed tomography
EF: Ejection fraction
MVV: Maximal voluntary ventilation
RUL: Right upper lobe
PV: Pulmonary Vein
